# Other primary headaches—thunderclap-, cough-, exertional-, and sexual headache

**DOI:** 10.1007/s00415-020-09728-0

**Published:** 2020-03-04

**Authors:** Anish Bahra

**Affiliations:** The National Hospital for Neurology and Neurosurgery, University College London (UCL), Queen Square, Box 80, London, WC1N 3BG UK

**Keywords:** Thunderclap headache, Cough headache, Exertional, Sexual, Coital, Orgasmic headache

## Abstract

This article reviews the disorders of thunderclap, cough, exertional and sexual headache. These are a group of paroxysmal and precipitated headaches, which often occur in bouts with prolonged remissions. Indometacin seems to be the most effective preventative. Each can occur in primary and secondary form. Thunderclap headache is the most frequently reported headache syndrome associated with a secondary pathology. Discussed are the complexities of whether all patients with thunderclap headache should have further investigation if timely computerised tomography is normal and, the relevance of abnormal imaging in these disorders, differentiating what is deemed to be secondary and managing the pain.

## Introduction

A primary headache is synonymous with a headache disorder having no clear correlating aetiology on examination of the patient nor on structural imaging. The terminology has evolved from ‘benign’ and ‘idiopathic’ to the current use of ‘primary’. The majority of individuals experience spontaneous and recurrent episodes of self-limiting pain. The International Classification of Headache Disorders (ICHD) has been successful in refining phenotypes which, based upon history alone, provides well-defined clinical syndromes [1] likely to respond to specific treatments.

Knowledge of the primary headaches comes largely from the work in the most prevalent disorders, namely tension-type headache, migraine, and cluster headache. This supports the concept that these disorders occur in those genetically predisposed [2–4] and, where the inherent pathology is in the central nervous system. When individuals first experience a headache, the concern is often about a sinister precipitating pathology [5]. However, in patients presenting with tension-type headache, or migraine with and without typical aura and a normal neurological examination, the prevalence of an underlying brain lesion is the same as that in an asymptomatic population [6, 7]. With more uncommon disorders, acquiring population-based data is much more challenging and the literature is confounded by publication bias.

Part I of the ICHD orders the primary headaches into four sections: migraine, tension-type headache, the trigeminal autonomic cephalalgias and a fourth groups of miscellaneous, largely paroxysmal headache disorders. Although the fourth section of the ICHD has been one into which miscellaneous disorders have been consigned the grouping does reflect syndromic and, pathophysiological alignment. Most of the syndromes are precipitated, paroxysmal and usually short-lived (Table 1). Moreover, the most consistent preventative treatment seems to be Indometacin. New daily persistent headache is the exception; whether this is indeed a distinct pathological entity remains to be determined.

**Table 1:** The other primary headaches [1]

Primary cough headache
Primary exercise headache
Primary headache associated with sexual activity
Primary thunderclap headache
Cold stimulus headache
External-pressure headache
Primary stabbing headache
Nummular headache
Hypnic headache
New daily persistent headache

This review will focus on primary thunderclap, cough, exertional, and sexual headache. Each disorder can occur in primary and secondary forms.

## Thunderclap headache

### Clinical syndrome

Thunderclap headache (TCH) describes a sudden severe explosive onset headache. The severity itself is not diagnostically helpful [8–10], it is the rapidity of onset, with pain developing from zero to maximum in seconds to minutes.

### Epidemiology

Landtblom has provided the only population-based data citing an incidence of 43 per 100,000 persons (> 18 years) per year of *all* sudden onset headache (within 10 s) [9] with primary TCH (PTCH) being cited as occurring in 38 per 100,000 persons per year.

### Primary and secondary thunderclap headache

It is best to understand TCH from a historical perspective. Isolated thunderclap onset headache is the most consistently reported presentation of a secondary headache, with the time frame of the pathology and onset of the headache supporting an association [5]. The term ‘isolated’ infers the headache to be in neurologically and systemically intact patients. The most dramatic presentation is subarachnoid haemorrhage (SAH); one third present with isolated headache. It is from this literature that our knowledge of TCH has evolved. The prevalence of spontaneous SAH is about nine cases per 100,000 people a year, thus a rare disease [11]. The disproportionate attention it has garnered lies in the high mortality. A meta-analysis of 33 studies reported a case-fatality rate of 8∙3–66∙7% (variability depending on country of data acquisition). Also reported was a 17% reduction in case-fatality over three decades, an observation supported in subsequent populations [12]. The improvement has been attributed to better management, primarily rapidity of access to medical care.

Of all spontaneous SAH, 85% are aneurysmal while 10% are perimesencephalic, thus venous and carrying a generally benign prognosis. The most definitive method of diagnosis is unenhanced computerised tomography (CT), which will show SAH in 99% of cases if imaged within 6 h of ictus [13, 14]. In those with negative CT, examination of cerebrospinal fluid (CSF) for altered blood products remains positive from 12 h to 2 weeks in 100% of cases [15–17]. The risk of angiography in lieu of CSF examination, or in late presentations, is detection of an incidental aneurysm and potential unnecessary and cost- ineffective intervention [18, 19]. It has been argued that using third generation CT scans to image patients within 6 h of ictus, the sensitivity and specificity for detecting *aneurysmal* subarachnoid haemorrhage is 100%. While the detection of subarachnoid blood on CT- negative patients is about 7%, the cause for the CSF xanthochromia in these patients runs a benign course [20, 21].

The existence of a benign form of thunderclap headache is supported by longitudinal observations of patients investigated with normal CT and CSF examinations who did not develop any subsequent adverse sequalae and continued to experience recurrent attacks (Table 3). Moreover, it was shown that clinically it is not possible to reliably differentiate those who have experienced a subarachnoid haemorrhage from those with subsequently normal investigations [8, 9, 22–25]. In both groups the onset of the headache is within seconds to a few minutes in most; in 20% escalation of severity can be greater than 5 min [8, 10]. There is no difference in severity, past history of headache, precipitating event (exertion or valsalva) nor additional neurological symptoms. Moreover all groups had previously experienced similar TCH. Clinical guidelines have been developed for diagnosing SAH in patients presenting with acute headache with almost 100% sensitivity but poor specificity of 13.6% [26].

Thunderclap onset headache has also been associated with a multitude of secondary pathologies. A systematic review of the literature from 2014 identified 119 causes in a total of 2345 cases reported in isolation, case series or cohorts [27]. The diagnoses relied on the combination of ‘sudden’ and ‘severe’. The definition of ‘sudden’ was not detailed and it was acknowledged that the majority had not used ICHD criteria. Within each pathological category are a number of likely incidental findings.

By far the largest contribution was primary headache in 459 cases, 213 primary thunderclap headaches, followed by primary sexual headache in 182, bath-related headache in 37 and exertional headache and combinations of the aforementioned. Three-hundred and ninety-eight cases were precipitated by cerebrovascular disorders, 206 from SAH, 46 from other sources of intracranial haemorrhage, venous and arterial thromboembolism, intracranial dissection, stroke, hypertensive encephalopathy and vasculitis. Included were 18 cases likely to be incidental findings, primarily unruptured cerebral aneurysms. One hundred and sixty cases of sudden and severe headache were reported in association with infection, 44% affecting the CNS and the remainder systemic with likely CNS involvement or, encompassed within the terminology of a ‘viral illness’ without further elaboration. One hundred and seventy-three patients were diagnosed with reversible vasoconstrictive ‘syndrome’ (RCVS). The largest contribution to the 119 non-vascular precipitants was from pituitary apoplexy in 43 cases and 32 related to alterations in CSF pressure. Less than 20 cases were related to the peripartum state and drugs.

The current ICHD of primary thunderclap headache defines the rapidity of onset as being within 1 min (Table 2). This will capture up to 75% of primary and secondary thunderclap headache, while altering this definition to escalation within 5 min will capture 95% of cases [8]. The subsequent headache, following the initial thunderclap, most frequently has clinical features consistent with migraine (Table 3).

**Table 2:** International classification of headache disorders [1]

Thunderclap headache	Abrupt onset, reaching maximum intensity in < 1 min Lasting for ≤ 5 min
Cough headache	Sudden onset Brought on by, and occurring only in association with, coughing, straining and/or other Valsalva manœuvres Lasting between 1 s and 2 h
Exercise headache	Brought on by, and occurring only during or after, strenuous physical exercise Lasting < 48 h
Headache associated with sexual activity	Brought on by and occurring only during sexual activity Either or both of the following: 1. Increasing in intensity with increasing sexual excitement 2. Abrupt explosive intensity just before or with orgasm Lasting from one minute to 24 h with severe intensity and/or up to 72 h with mild intensity

The assumption of a primary disorder is recurrence (> 1 attack) with no evidence for an alternative secondary precipitating pathology

**Table 3:** Thunderclap headache

Publication	N	Mean Follow-up (years)	Past TCHA %	Recurrent TCHA %	Subsequent primary HA %	Prior primary HA%	Precipitant to onset	Duration	Additional features (%)
Wijdicks 1988 [22] PTCHA	71	3.3	10	17	44	–	Cough 7%, sex 4%, other exertion 17%, light tasks 72%	8–24 h median Range 1 h to 1 week	Vomiting (38), diplopia (3), stiff neck (14), dilated pupil (1)
Harling 1989 [23] PTCHA	14	1.5 to 2.5 (no mean given)	–	–	93	28	Exercise, weights, sex 21%	< 2 h 0% > 2 h 79%	Vomiting (28) (P < 0.02), Neck stiffness (57) Photophobia (64) Loss of consciousness (14)
Harling 1989 SAH	35		–	–	–	43	Exercise, weights, sex 28%	–	Vomiting (72) Neck stiffness (80) Photophobia (57) Loss of consciousness (34)
Markus [24] PTCHA	18	1.7	25	25	50	38	*N* (19%) Sex 1 Straining 1 Lifting 0 Diving 1	–	Nausea (88) Vomiting (44) Photophobia (50) Collapse (6)
Markus 1991 SAH	37	–	60	–	–	–	N (18%) Sex 1 Straining 0 Lifting 1 Diving 1	–	Nausea (60) Vomiting (68) Photophobia (5) Collapse (16)
Linn 1998 [8] PTCHA	42	Data from first presentation only	14	–	–	57	Exertion/Valsalva 22%	–	Nausea (76) Vomiting (43) Transient loss/clouding of consciousness (16) Transient focal symptoms* (22) Seizure 0%
Linn 1998 SAH	37		19	–	–	38	Exertion/Valsalva 50%	–	Nausea (76) Vomiting (69) Transient loss/clouding of consciousness (26) Transient focal symptoms* (33) Seizure (7)
Landtblom 2002 [9] PTCHA	101	1	29	24	–	28 Migraine 25 TTH	Exertion/Valsalva 21% Sexual TCHA 9%	–	Nausea (91) Neck stiffness (61) Paresis (13) Impaired consciousness (17) Unconsciousness (17) Photophobia (9) Blurred vision (4) Scintillation scotoma (0) Diplopia (0)
Landtblom 2002 SAH	23	–	17	–	–	22 Migraine 9 TTH	Exertion/Valsalva 17% Sexual TCHA 9%	–	Nausea (61) Neck stiffness (10) Paresis (3) Impaired consciousness (9) Unconsciousness (4) Photophobia (4) Blurred vision (4) Scintillation scotoma (7) Diplopia (2)

*PTCHA* Primary thunderclap headache, *SAH* Subarachnoid haemorrhage

^*^Transient neurological symptoms—double vision, speech arrest, sensory phenomena or weakness in the face or limbs (unilateral or bilateral), and difficulties with walking

In summary, TCH is the most common headache syndrome associated with a secondary precipitating pathology. The majority will have PTCH [9]. Primary and secondary thunderclap headache, however, cannot be reliably differentiated clinically thus, all patients should be investigated. Within 6 h of ictus, it is likely that aneurysmal SAH can be identified by CT alone. However, late presentations and less common causes, will require further investigation.

## Cough headache

The lifetime prevalence of primary cough headache is reported as 1% [28]. The disorder has a male predominance.

Table 2 gives the current definition for cough headache and Table 4 the characteristics of published cohorts.

**Table 4:** Primary cough headache

Publication	*N*	Mean Age	Trigger *	Character of pain	Attack Duration	Bilaterally (%)	Features	Attack Frequency	Disease Duration (months)	Treatment response
Symonds 1956 [31]	21	55	Valsalva Head rotation	Severe Bursting	2–10 min	All	–	–	18–36	None given
Pascual 1996 [32]	13	67	Valsalva	Mod/severe Sharp/stabbing	Secs to < 30 min	92	–	One-several/day	2–24	6 treated with Indometacin 75 mg—all responded
Ozge 2005 [34]	20	45**	Cough headache only reported	Mod/severe Sharp/stabbing	1–30 min	90	Nausea 5% Dizzy 10%	10 days/month	–	
Pascual 2008 [33]	28	60	Posture Valsalva	Electrical Explosive Pressing	Secs to > 1 min	39	Dizzy 14%	–	1–42	9 treated treated with Indometacin 50-100 mg—all responded −5 months max needed
Chen 2009 [30]	74	61	Bending Valsalva	Mild-severe Explosive Dull/Pulsatile	1 min to 2 h	67	Nausea 10% Vomiting 1% Photophobia 5% Phonophobia 11%	-	6–24	72.7% treated with Indometacin 75 mg responded

^*^Vasalva: Coughing, sneezing, straining ( often at stool), laughing, lifting, bending

^**^Primary and secondary together

Cough headache is precipitated (rather than *aggravated,* as occurs in migraine) by a valsalva manoeuvre such as coughing, sneezing, bending, straining and, laughing (with genuine mirth) [29]. The pain is immediate, short-lived, usually bilateral and with a paucity of additional features. The periods of time during which individuals are symptomatic does tend to be self-limiting, lasting months or a few years at most [30–34].

Rooke’s original cohort of 103 patients, observed over three years, although titled ‘Exertional headache’ was a combination of valsalva related headache and exertional headache [35]. Most of the precipitants were valsalva manoeuvres, except for one which was running. Of these, 30 went into remission within 5 years and 73 improved or were free from headache at 10 years. Ten had an abnormality on imaging, an Arnold-Chiari malformation (ACM) in 3, platybasia in one, basilar impression in 2, subdural haematoma in 2 (non-acute), a parietal glioma in one and cerebellar haemangioendothelioma in one. In the patient with a known subdual collection over 20 years symptoms improved spontaneously without intervention. The glioma was detected 3 years after the onset of symptoms. The majority operated on had either resolution or improvement of the disorder.

Valsalva can precipitate other headache phenotypes, for example valsalva-induced cluster headache, paroxysmal hemicrania, hemicrania continua [36–38] and as described, thunderclap headache (Table 3). Rozen reported seven patients who presented with valsalva precipitated new daily persistent headache, five of whom had characteristics of migraine.[39]. Some patients may need combination therapy with treatments effective for each headache phenotype.

### Treatment

The most consistent treatment is Indometacin, effective over a dose range between 25–250 mg. Mathew first reported the response to Indomethacin in a double-blind placebo controlled manner in two patients with cough headache resistant to other tried preventatives [40]. Response was achieved in 1–4 weeks and maintained over an 18 month follow-up period.

The aim is to achieve an optimal dose which successfully suppresses the symptoms and then periodically reduce the dose to see whether the symptoms have become quiescent [30, 32, 33].

There are also anecdotal reports of treatment responses to acetazolamide [41], naproxen [42], propranolol [43] and lumbar puncture [30, 44]. The latter involved removal of 40 ml of cerebrospinal fluid. Constituents were normal.

### Primary and secondary cough headache

Despite the prevalence data of 1%, the published cohorts of cough headache remain small. The assumption is that this is because the disorder tends to be self-limiting and remissions prolonged. The primary cases tend to be more common than secondary cases (Tables 4 and 5). There is a preponderance of published posterior fossa lesions purported to be secondary causes, with ACM I disproportionately represented [45]. The natural history of ACM I in both asymptomatic and symptomatic patients (which includes those with a syrinx) is benign and without progression in the majority [46]. Analysis of ACM I cohorts show that the prevalence of headache is generally in line with population prevalences, although that for cough headache has been reported higher than the 1% population prevalence [47–49]. Although outcome of headache in those operated upon is reported to be good this is based on a relatively limited follow-up period. In one cohort of 96 patients 75% had resolution or improvement, the follow-up period was a mean of 3.6 years; the 30 day post-operative complication rate was 27% and included one death [49]. In another cohort a comparison was made between conservative (68 patients) and operated groups (109 patients). The improvement in cough headache was 40% and 94.6%, respectively, and for migraine 61.5% and 92.9%. The mean follow-up period for the conservative group was 4.8 years and that of the operated group only 1.3 years which, does not take into account the natural history of the primary disorder [47]. Moreover, none of the reports can account for the significant impact of surgical placebo [50]. Risks of intervention include wound sepsis, meningitis, stroke, CSF fistula and hydrocephalus [46].

**Table 5:** Secondary cough headache

Publication	Primary (*n*) (mean age years)	Secondary (*n*) Abnormal scan (mean age) *	Secondary (*n*) + Abnormal signs related to lesion on scan	Intervention	Follow-up duration (months)	Resolution following intervention	Resolution without intervention	Drug responders	Pathologies (*n*)
Symonds [31]	21 (55)	6 (52)	2	4 ( one operative death)	UK	1—air encephalogram and deep-xray treatment 1—air encephalogram	1—Spontaneous 4 year remission One-symptoms started after intervention^a^	NA	Posterior fossa meningioma Midbrain cyst Pagets with basilar impression (2) Post acoustic neuroma removal^a^
Pascual [32]	12 (67)	17(39)	UK 14 had symptoms or signs	8–C1-3 laminectomy (one for headache alone) –	UK	7 ‘improved’	‘persisting’—duration UK	None to ‘analgesics and antimigraine treatments’	All ACM I—5 with syringomyelia
Ozge [34]	20 (45)**	12	None documented	No intervention	Cross-sectional	NA	All resolved after treatment with Indomethacin	Analgesics partly effective Indomethacin 75–150 mg for 6 weeks (one also methysergide for concomitant migraine)—all resolved after withdrawal	3 ACM I Details of the rest unclear
Pascual [33]	28 (60)	40 (44)	No abnormal signs documented	9 (all for ACM I)	UK	9		5 treated with Indomethacin—no response	ACM I (32) Other posterior fossa lesions (8)
Chen [30]	74 (61)	9 (55)	3 ( ataxia and dysmetria)	4	51.4	4	0^b^	Indomethacin 37.5%	Cerebellar mass + Hydrocephalus (3 meningioma; 1 metastases) ACM I (2; up to 5 mm descent)^b^ Diffuse brain metastases (1) Subdural haematoma(1) Mucous retention cyst in the sphenoid sinus

*ACM* Arnold-Chiari malformation

*UK* Unknown

^*^No other abnormal tests

^**^Primary and secondary together

Secondary cough headache has also been reported to respond to Indometacin [51, 52]. In the case report of Buzzi, a 54 year-old patient presented with a 10 year history of cough headache as an isolated presentation. She was found to have an abnormal examination and imaging showed a Chiari 1 malformation and a syrinx extending from C7-T7. The episodes of Valsalva-precipitated headache responded to Indomethacin 25 mg bd.

This case highlights the importance of adhering to the Hippocratic principle to ‘Do No Harm’. This is particularly pertinent in the cases of patients presenting with valsalva headache, normal neurological examination and a Chiari I. It is clear that not all patients with a Chiari I develop cough headache [45]. The onset of the Valsalva headache occurs in the 5–6th decades while the malformation is considered congenital. Conservative management of paediatric populations, looking at natural history, suggest that the outcome in most patients is benign [53, 54]. There are occasional cases where there is both worsening and improvement; of the latter 3 of 147 patients had spontaneous improvement of the associated syrinx [54]. In Symond’s original cohort of cough headache patients, one patient developed symptoms after successful treatment for an acoustic neuroma. Another gave a 10 years history of cough headache with a 4 years history of spontaneous remission; when seen at 10 years she was found to have basilar compression from Paget’s disease with abnormal cranial nerve examination and a mild spastic paraparesis [31]. There is a single case report of cough headache, without precipitating thunderclap headache, associated with bilateral acute parietal infarcts and reversible vasoconstriction [55]. Suppression of the headache was achieved with the use of an antitussive, benproperine.

Thus, not only can secondary cough headache respond to medical treatment, but the disorder can also go into spontaneous remission, occur coincidentally and be precipitated by surgical intervention. Accordingly, it is proposed that any surgical intervention should be considered for neurological *progression* at a point where the risks of surgical intervention are warranted. In isolated cough headache, whether primary or secondary, preventative treatment with Indomethacin can be considered.

## Exertional headache

Population-based prevalence of exertional headache has varied from 1% [28, 56] up to 12.7% [57, 58] with more recent studies citing a female preponderance [56–59].

Table 6 gives the clinical characteristics of exercise-related headache. The exertional precipitant is usually a sustained strenuous effort which precipitates the headache during or after exertion. The attack can last minutes to hours, with the ICHD adopting an upper limit of 48 h to capture the majority of attacks. The subsequent headache, which is triggered, is usually throbbing and can be accompanied by features usually seen in migraine, albeit at lower frequencies than in migraine. In Hanashiro’s small cohort, 17% and 23% also experienced valsalva and sexual headache, respectively [56], while in Chen’s cohort of over 500 sufferers 47% had also experienced valsalva related headache. Silbert reports that in his cohort of 45 patients with benign sexual headache 27 (60%) also reported experiencing exertional headache [60]. Bougea reported on three patients with comorbid exertional-, cough-, and sexual headache [61].

**Table 6:** Primary exertional headache

Publication	*N*	Age years	Trigger *	Character of pain	Bilateral (%)	Features	Attack Duration	Attack Frequency	Disease Duration	Other headache
Pascual 1996 [32]	16	Mean 24	Prolonged exercise	Pulsating	56	Nausea Photophobia	Minutes-2 days (median 4 h)	1/day—1 every 2 months	1/day—1 every 2 months	**–**
Sjaastad 2002 [57]	202	18–65 Age of onset < 30	Strenuous hard activity Skiing, Competitions ‘Heavy’/‘maximal’ exercise/gymnastics, Swimming, Running	Throbbing	Mostly	Nausea 8% Vomiting 0.004% Photophobia 5%	Few mins-one day	10- multiple episodes a day	10 years	Migraine 46%
Chen 2009 [59]	596	Median 13–15	Strenuous activity	Throbbing	51	Nausea 29% Vomiting 7% Photophobia 18% Phonophobia 27%	< 1 h	–	–	Migraine 35% Valsalva 47%
van der Ende-Kastelijn 2012* [81]	1810	Median 31–45	Cyclists only	Dull 42% Throbbing 23%	67	Photophobia 47% Neck pain 40% Phonophobia 35% Nausea 15% Vomiting 3%	1–6 h 30% To next morning 32%	Median once/month	–	Migraine 10% Tension-type headache 24% Cluster headache 1.4%
Hanashiro 2015 [56]	30	Mean 40	**–**	Throbbing	77	Not given	5 min–12 h		Mean 4 months	Migraine 67% Valsalva 17% Sexual 23%
Tofangchiha 2016 [82]	38	Mean 22	*Iranian military conscripts training* Aerobic exercise Weight lifting Football/Volleyball Walking/Inclines Routine homework	Pulsating 47.4% Compressive 44.7% Indeterminate 7.9%	73.7 21.1 (both)	Not given	< 5 min -31.2% 5-60 min -39.5% 1–24 h–42.1% > 24 h–5.3%	Out of 10 sessions < 2–44.7% 2–5—36.8% > 5–18.4%	–	–

The cohort of Rabiee is not included because of the disparity in percentages and numbers given in the text and tables. In addition the number of individuals with different headache characteristics in the tables seems to be from the whole sample of 2076 from which the 152 patients with exertional headache were identified (58)

There is little information about the natural history of the disorder. That available and that from duration of treatment required, suggests the disorder occurs in self-limiting bouts as brief as a month up to a few years.

### Treatment

Diamond reported on the complete resolution of symptoms with Indomethacin in 13 of his 15 patients within 1–4 weeks; medication was withdrawn after three to 12 months with all but one patient remaining asymptomatic, suggesting natural remission [62]. In Pascual’s series of 16 patients 4 seemed to respond to pre-emptive ergotamine, one patient with regular attacks to propranolol and one each to Indomethacin and Flunarizine [32]. Five of nine patients in a later cohort, responded to prophylactic nadolol or propranolol over a period ranging from two to six months [33]

### Primary and secondary exertional headache

The main forms of exertional headache associated with a secondary pathology are those where exertion precipitates a TCH. Management remains as detailed in the section TCH.

## Cardiac cephalgia

Cardiac cephalgia is a rare exertional headache secondary to cardiac ischaemia and responds to treatment of the cardiac ischaemia. The entity is now recognised in the Appendix of the ICHD. A recent 2017 review of the case reports (*n* = 35) [63] found that there was a male preponderance with mean age of onset of 62 years. Half of the cases were precipitated by exertion, sexual activity and emotional fluctuation. The clinical syndrome was of a bilateral or unilateral headache, with or without typically migrainous features but severe in 33 of the patients. Only half presented with characteristics associated with cardiac ischaemia, such as chest pain or tightness, palpitations and dyspnoea. The headache settled with rest and could be eased by anti-anginal treatment such as nitroglycerine spray. This is a particularly useful indicator, given that in headaches of a similar phenotype, namely migraine and tension-type headache, nitroglycerine precipitates the headache [64]. Most cases had an abnormal baseline ECG and elevated cardiac enzymes. Of those who had indicators for cardiac ischemia, three had a normal baseline ECG, one a normal exercise stress test and two normal angiography.

## Sexual headache

The population prevalence of sexual headache is cited as 1–1.6% [28, 65]. There is a male preponderance and familial occurrence, one report in mother and daughter [58] and another in four sisters suffering from sexual headache [66]. Primary headache associated with sexual activity is divided into pre- and post-orgasmic forms (Table 2).

***Preorgasmic headache*** is a headache which gradually increases in severity towards orgasm and occurs in about 20% of cases of sexual headache. Pornography precipitated headache has been reported in a 40 year old man who experienced pre-orgasmic headache within 10 min of watching pornography, only on the internet. The pain subsided when the patient stopped watching and responded to pre-emptive Indomethacin, taken 15 min prior to watching [67].

***Orgasmic headache*** is a sudden onset severe pain (thunderclap onset) immediately before or at orgasm and is the mode of presentation in about 80% of cases.

Sexual headaches are not experienced with every sexual encounter [32, 60, 68, 69]. The attacks of headache tend to be short-lived from a few hours, infrequently longer (Table 7). Accompanying symptoms are uncommon, the most frequently cited being nausea. In Frese’s cohort of 51 patients, the sexual headache most commonly occurred with sexual activity with the usual partner (94%), but also during masturbation (35%), with a new partner (14%), and only during an extramarital affair in one patient. Twenty patients (40%) could terminate the headache by stopping sexual activity. Twenty-six patients (51%) could ease the headache by taking a more passive role during sexual activity. In five patients sexual headache occurred only with specific sexual practices.

**Table 7:** Primary sexual headache (sexual intercourse and masturbation)

Publication	*N*	Age years (mean)	Evolution	Character of pain (*n*)	Bilateral (%)	Features (*n*)	Attack duration	Attack frequency	Disease duration	Other headache	Successful treatment
Lance [68]	21	40	Gradual 3 Sudden 16 (Rest from sleep/unknown)	Dull/tight (4) Throbbing (5) Explosive (3) Abrupt severe (7) Unknown (2)	86	None given	3 min–4 h (mild residual pain 48 h)	Several occasions in succession–then remissions for months—years	At first consultation 1 month–3 years	Migraine 19% Exertional headache 9%	–
Ostergaard [73]	26	Median 32	Gradual 2 Sudden 19 Sudden + postural 5	Gradual–dull Sudden–explosive and throbbing initially, dull on subsequent resolution	Mostly	None (blurred vision in one)	In 4 patients 5–15 min In 22 patients severe for 5–15 then gradual resolution over 1–24 h ( 10 were motion sensitive)	In the 22 patients–recurrent attacks—over 6 weeks in 18 patients and up to 6 months in 4 patients	Single bout in 50% In those with recurrence remission up to 10 years between bouts	Migraine 19% Tension-type headache 27% ( more likely to have recurrent attacks of sexual headache)	–
Pascual [32]	13	41	Sudden 13	Explosive & pulsating	77	None	1 min- 3 h	3/day–1/month	6 days–18 months	Migraine 31% Tension-type headache 15%	Nadolol, propranolol
Frese [72]	40	34	Sudden (explosive)	Throbbing 50%	68	Nausea 13 Nausea + Photophobia 1 Mood disturbance 4 Dizziness 7	10 min–2 h (mild thereafter 45 min–12 h)	Bouts**†** 2 days–18 months (2–50 attacks per bout) Mean remission 22 months 82% single bout ( 3 year follow-up) 2 bouts 15% Chronic (*n* = 15)–7 went into remission		Migraine 25% Tension-type headache: Episodic 35% chronic 10% Exertional headache 29% Cluster headache 2% Trigeminal neuralgia 2%	Indomethacin Betablockers
Frese [72]	11	39.5	Gradual (dull)	Throbbing 36%	64		10 min–6 h (mild thereafter 25 min-12 h)				
Donnet [83]	20	49.3	Immediate onset (1–5mins) 16	Pulsatile 35% Compression 30% Explosive 30% Stabbing 5%	65	Nausea 7 Phonophobia 4 Photophobia 3	3 min to 5 h (mean 20 min)	–		Exertion/Sport 40% Valsalva 20% Migraine 50%	–

^**†**^At least two attacks occurring in at least 50% of sexual activities and then none for at least 2 weeks despite continuing sexual activities

Orgasmic aura without headache has been reported in two patients [70]. A 23 year-old woman with a history of migraine, experienced vertigo, oscillopsia and a perception of weakness in the legs at the onset of orgasm, with full recovery by 45 min. Symptoms responded to nifedipine 10 mg twice a day. A 33 year-old man experienced vertigo at orgasm, sometimes accompanying fortification spectra lasting 10–20 min. Pre-emptive diclofenac, an hour before sex, was effective at preventing the symptoms. Independently he could experience vertigo for 10 min followed by a migraine headache, lasting up to 6 h.

Particularly given the male preponderance, the prevalence of migraine is higher than the population prevalence, up to 30%. In a cohort of 100 migraine sufferers and 100 controls, none of the latter group had a history of sexual headache while 5% of those with migraine had also experienced sexual headache [71]. Comorbidity with exertional headache was reported in three of the five cohorts presented in Table 7, while only one cohort reported comorbidity with valsalva-associated headache, in 20%.

As with the other precipitated primary headaches, primary sexual headache seems to occur in bouts with prolonged remissions. The latter is often several years in duration. In Frese’s cohort, 82% had a single bout over a three year follow-up period [72]. Ostergaard’s follow-up of 26 patients spanned 6 months to 14 years (median 6 years). Thirteen patients had recurrent bouts of sexual headache interspersed by remissions lasting up to 10 years [73].

### Treatment

The most consistently reported responses in sexual headache are for Indomethacin and betablockers, most commonly cited propranolol, but also metoprolol and nadolol [32, 42, 66, 69, 74, 75]. Raskin recommended the use of pre-emptive Indomethacin 50 mg after dinner [76], successful in four out of five patients. Prophylactic success with Indomethacin, oral treatment 25 to 100 mg given 30–60 min prior to sexual activity, has corroborated Raskin’s experience [77, 78]. Frese reported benefit with triptans for the headache precipitated by sexual activity if attacks usually lasted longer than 2 h. The paper also reported on the preventative benefit of rizatriptan, almotriptan and sumatriptan, 30 min before sexual intercourse [77].

### Primary and secondary sexual headache

The majority of patients with sexual headache present with thunderclap onset headache and therefore will need to be managed for a possible secondary precipitant. Lundberg reported on 4–12% of patients, confirmed to have subarachnoid haemorrhage, presenting with sexual thunderclap headache [79]. Headache secondary to spontaneous intracranial hypotension, precipitated by sexual intercourse, is no longer part of the classification for primary sexual headache but is a reported precipitant in spontaneous intracranial hypotension [80].

## Summary

Thunderclap, cough, exertional and sexual headache can occur as both primary and secondary headache disorders, the primary headaches predominating. These disorders can be viewed as modes of onset of a headache, with the subsequent headache most commonly having characteristics of migraine or, tension-type headache. Although thunderclap headache can occur spontaneously, it can also be triggered.

This group of disorders tend to occur in bouts which are self-limiting, in the majority lasting months or a few years. Remissions periods can be prolonged over many years. A regular pattern of bouts and remissions, as seen in cluster headache, is not characteristic. About half are reported to have had a single bout only, during the follow-up periods reported. Patients tend to be more likely to have other comorbid headache disorders than would be expected based on population prevalence. The most consistent treatment responses across the group are seen with Indometacin.

The infrequency with which each disorder is seen, based upon prevalence but also remission periods, makes it more difficult to address the issue of what proportion with the isolated headache syndrome have a primary headache or are secondarily precipitated. More specifically, who should be imaged? It is clear that all patients presenting with a thunderclap headache, whether precipitated or not, will need investigation. The issue is more difficult with cough, exertional and sexual headache because of the risk of identifying an incidental lesion. This is more so because these disorders are self-limiting, thus successful case reports of intervention may simply indicate natural remission. Moreover, the primary and secondary headache phenotypes are identical, therapeutically respond similarly to medical treatment and, intervention for any presumed precipitant does not necessarily result in resolution of the headache. The report of two familes with sexual headache, suggests that, as with the other primary headache disorders, these disorders may also occur in those genetically predisposed. Until there are larger published cohorts to inform whether each phenotype (other than TCH) has a higher risk of identifying a brain pathology than a matched asymptomatic population, those with new onset symptoms are likely to be imaged. A pragmatic approach would be to treat any abnormality and the headache independently. It would seem appropriate to reserve intervention based upon neurological progression related to the lesion and *not* the pain alone.
